# Uneven Effects of Sleep Apnea on Semicircular Canals and Otolithic Organs

**DOI:** 10.3389/fneur.2022.819721

**Published:** 2022-02-16

**Authors:** Xin-Da Xu, Bin-Jun Chen, An-Rong Sun, Qing Zhang, Ying Cheng, Dong-Dong Ren, Jing Yu, Hui-Ping Luo

**Affiliations:** ^1^ENT Institute and Department of Otorhinolaryngology, Eye & ENT Hospital, Fudan University, Shanghai, China; ^2^NHC Key Laboratory of Hearing Medicine Research (Fudan University), Shanghai, China; ^3^Department of Otorhinolaryngology Head and Neck Surgery, Xinhua Hospital, Shanghai Jiao Tong University, Shanghai, China; ^4^Department of Otorhinolaryngology, Head, and Neck Surgery, Second Affiliated Hospital of Xi'an Jiao Tong University School of Medicine, Xi'an, China; ^5^The Therapy Center of Sleep-Disordered Breathing, Eye & ENT Hospital, Fudan University Shanghai, Shanghai, China

**Keywords:** vestibular-evoked myogenic potential (VEMP), obstructive sleep apnea (OSA), vestibular function, otolithic organ, semicircular canal

## Abstract

**Objective:**

This study aimed to explore how obstructive sleep apnea (OSA) affects the function of each vestibular organ and to identify the correlations among them.

**Methods:**

A prospective study was conducted involving 32 healthy controls and 64 patients with OSA. The objective detection methods of the utricle and saccule are vestibular-evoked myogenic potentials (VEMPs). A combination of the caloric test and video head impulse test (vHIT) was used to comprehensively evaluate the objective function of semicircular canals.

**Results:**

Elevated thresholds (*p* < 0.001), decreased waveform amplitudes (*p* < 0.001), prolonged first wave latencies (*p* < 0.001), and shortened first interpeak latencies (*p* < 0.001) were observed in both ocular VEMP (oVEMP) and cervical VEMP (cVEMP). A significant difference was found in the caloric test comparison (χ^2^ = 4.030, *p* = 0.045) but not in the vHIT. The intergroup comparison of normal rates among the VEMPs, caloric test, and vHIT groups showed a significant difference (*p* < 0.001).

**Conclusion:**

The impairment of vestibular function in patients with OSA was uneven and biased. More attention should be given to vestibular dysfunction in the diagnosis and treatment of OSA.

## Introduction

Patients with obstructive sleep apnea (OSA) repeatedly suffer from soft tissue collapse of the upper airway during sleep, resulting in a nocturnal low oxygen desaturation condition at night, which, in turn, leads to sympathetic nervous system hyperactivity and impaired neurocognitive function. Studies have shown that the cochlea and its neural pathways may be adversely affected by apnea and hypoventilation (1), with which the vestibular system may share the same supply of blood. The impairment of the vestibule can lead to a variety of serious life problems (2), and it is considered a major public healthcare problem (3). Therefore, studying the status and change pattern of vestibular function in patients with OSA is of great significance.

The vestibular organs, which consist of the utricle, saccule, the lateral semicircular canal (LSC), the superior semicircular canal (SSC), and the posterior semicircular canal (PSC), and their corresponding neuromuscular reflex constitute the vestibular system. The objective detection methods of the utricle and saccule are vestibular-evoked myogenic potentials (VEMPs), namely, ocular VEMP (oVEMP) and cervical VEMP (cVEMP) (4, 5). The objective detection methods of semicircular canal function include the caloric test (6) and video head impulse test (vHIT) (7, 8). The vHIT shows greater advantages in precisely positioning the semicircular canals with pathological changes. The combination of the caloric test and vHIT can comprehensively reflect the objective function of semicircular canals at different frequency ranges of stimulation (7).

Related literature is limited and researchers still have diverging views on the effect of OSA on vestibular function. Some researchers reported a significant effect (9), while others thought that there was no effect at all (10). In addition, studies on semicircular canal function in patients with OSA are rare, and this inspired our interest in exploring this topic. In this study, a battery of tests that included oVEMP, cVEMP, caloric test, and vHIT was used. It is aimed to explore how OSA affects the function of each vestibular organ and identify the correlations among them.

## Materials

### Subjects

The data were collected from December 2020 to September 2021. Patients were diagnosed with OSA at the Therapy Center of Sleep-disordered Breathing of the Eye Ear Nose & Throat Hospital of Fudan University and enrolled in this prospective clinical study.

The subjects were 20–50 years in age, had no complaints of either vestibular symptoms or disorders before, and they had no history of ear disease, neurological disease, hypertension, diabetes, or other chronic conditions. Otoscopy and tympanogram confirmed that the eardrums and the middle ear were normal. Then, oVEMPs, cVEMPs, the caloric test, and vHIT were recorded and analyzed. Healthy sex- and age-matched volunteers who had no symptoms of snoring as a control group were also recruited and underwent a full set of examinations as the subjects.

This study was approved by the Institutional Review Board of the Eye Ear Nose and Throat Hospital of Fudan University [(2020)2020139]. Each patient and control subject provided written informed consent to participate in this study.

### Polysomnography

An overnight PSG test (Embla N7000, Embla, USA) was conducted. None of the eligible participants had a history of taking sedatives before the test. The details of PSG have been described in a previous study (11) and meet the criteria of the American Academy of Sleep Medicine (AASM) Scoring Manual 2.4 (12). OSA was defined as an apnea hypopnea index (AHI) of equal to or more than 5/h during sleep in patients with symptoms of the disorder. According to AHI severity, patients were classified as mild (5/h ≤ AHI < 15/h), moderate (15/h ≤ AHI < 30/h), and severe (AHI ≥ 30/h) OSA (12).

### Vestibular-Evoked Myogenic Potential Testing and Analysis

Vestibular-evoked myogenic potentials were performed in a soundproof examination room. A 500-Hz tone burst (rise/fall time = 2 ms; plateau time = 2 ms) was used. The Bio-Logic Navigator PRO (Natus, USA) was used to amplify the electromyographic signal. The bandpass filter was set at 10–1,500 Hz and the responses to 120 stimuli were averaged for each run. The ACS was transmitted *via* a calibrated earphone. To check whether VEMPs could be elicited in the subjects, a stimulus level of 125 dB sound pressure level (SPL) was used as the default starting intensity. The stimulus intensity was decreased or increased in steps of 5 dB SPL depending upon the presence or absence, respectively, of VEMPs.

A brief description of the testing process (5) is as follows. Each subject was tested in a supine position. During oVEMP testing, an active electrode was placed 1 cm below the lower lid in line with the pupil of each eye, a reference electrode was placed below the active electrode, and a grounding electrode was placed on the midline of the forehead. The interelectrode resistances were confirmed to be below 5 kΩ. Each subject was asked to look upward upon hearing a sound from the inserted earphone (13). For the cVEMP testing, an active electrode was placed in the middle of the sternocleidomastoid muscle, a reference electrode was placed above the sternoclavicular joint, and a grounding electrode was placed on the midline of the forehead. The interelectrode resistance was confirmed to be <5 kΩ. When each subject heard tone burst through the inserted earphone, she or he was instructed to raise the head off the pillow to activate the sternocleidomastoid muscle (SCM) (14). The muscle contraction fluctuation of cVEMPs has not been corrected because it was not available to monitor in our lab. Thus, the VEMPs responses were obtained.

A stimulus level of 125 dB SPL was used to check whether subjects were able to elicit VEMPs. VEMPs were considered to be present when reproducible short-latency biphasic waveforms were elicited. Unrecognizable waveforms or unrepeatable waveforms were considered the absence of VEMPs. The oVEMP and cVEMP response rates (%) for each group were calculated (response rate = *N*_present_/*N*_total_ × 100%). Normal VEMP results were defined according to the calibration of equipment in our laboratory: The normal values (mean ± 2SD) of threshold were 110.91 ± 6.57 dB SPL (oVEMP) and 107.34 ± 9.34 dB SPL (cVEMP) in our lab. Since the stimulus intensity would decrease or increase in the step of 5 dB SPL, then 105–115 dB SPL (oVEMP) and 100–115 dB SPL (cVEMP) were taken as the normal ranges of the threshold. The normal ranges of VEMPs amplitude were set in quartiles (7.05–22.05 μV in oVEMP and 119.40–331.46 μV in cVEMP). In our daily clinical work, 7 and 120 μV are used as the minimum normal value for oVEMP and cVEMP, respectively. In the clinical work of our hospital, if the examination results are outside the normal range, the patient would receive a retest after a half-hour rest, or reschedule an appointment after adequate sleep. Only if the results of two times tests were both outside the normal range, the VEMP results would be confirmed as abnormal, and the patient could be recommended to make an appointment at the Vestibular Center of our hospital for further examination to verify whether there were vestibular diseases or not. The normal rate (%) = *N*_normal_/*N*_total_ × 100%.

The lowest stimulus intensities were acquired as the VEMP thresholds (dB SPL) when a series of repeatable biphasic waveforms were observed and recorded. The amplitudes and latencies of the oVEMPs and cVEMPs were measured at a stimulus level of 125 dB SPL. Parameters, namely, the p1 and n1 latencies (ms), interpeak latencies (ms), and amplitudes (μV), were recorded. In the oVEMPs, the latencies of n1 and p1 were determined between 0 ms and the corresponding maximal peaks of the waveforms. The interpeak latency was defined as the absolute value of the elapsed time between n1 and p1. The amplitude was defined as the vertical distance between the peaks of the n1 and p1 peaks. Similarly, in the cVEMP, the threshold, p1 latency, n1 latency, p2 latency, p1-n1 interpeak latency, n1-p2 interpeak latency, p1-n1 amplitude, and n1-p2 amplitude were recorded.

The asymmetric ratios (AR) of amplitudes were calculated: AR = (|amplitude of one side – amplitude of the other side | / | amplitude of one side + amplitude of the other side |) × 100%. The normal value of our laboratory is <28%.

### Caloric Testing and Analysis

The caloric tests were performed in a darkroom using a GN Otometrics Type 1068 air irrigator with an airflow of 8 l/min at 50 and 24°C within 60 s, first in the right ear and then in the left ear. The interval between successive irrigations was 10 min. The maximum slow phase velocity (SPV) was determined using a GN Otometrics Type 1068 system. The upper normal limit of vestibular asymmetry (canal paresis, CP) was 25%. Bilateral canal paresis was considered to be present if the total response from both sides was <15°/s. The canal paresis was considered abnormal in this study, and normal rates were calculated (The normal rate (%) = *N*_normal_/*N*_total_ × 100%).

### vHIT Testing and Analysis

The vHIT was conducted using the Otometrics ICS Impulse A/S Taastrup equipment from Denmark and performed in a well-lit room. The subjects were given a special spectacle frame, which was lightweight and had a built-in camera that tracked their pupil movements. To perform the calibration, subjects were asked to keep their heads still and view the alternating laser spot on either side of the target placed 1 meter in front of them. The subjects then provided a slow sinusoidal motion of the head while keeping their gaze on the target. After calibration, subjects were instructed to keep their gaze fixed on a target point that was maintained according to the height of the participant at all times, even in head thrust. On each of the planes, a total of 15 times of head thrusts were carried out at an angle of 10–20° in random order. For the vertical canal test, the head rotates 30° to the left or right while maintaining a focus on the target, and then the head thrust is applied on the corresponding semicircular canal plane. Semicircular canal dysfunction was defined as a reduction in the angular vestibulo-ocular reflex (aVOR) gain value. For gain values, cutoff values of 0.8 (LSC) and 0.7 (SSC and PSC) have been proposed to distinguish abnormal gains (normal rate = *N*_normal_/_total_ × 100%) (15).

### Statistical Analysis

The data were analyzed using IBM SPSS Statistics software version 19.0.0 (USA). The chi-squared test was used for the comparison of the response rates of VEMPs and normal rates of each parameter. In the multiple comparisons of parameters, if the variance is homogeneous, then ANOVA was adopted; if the variance is not homogeneous, then the H test is adopted. A paired *t*-test was used to compare thresholds, latencies, interpeak latencies, and amplitudes. The Spearman's rank correlation test (correlation between parameters) was performed in R version 3.5.3. *p* < 0.05 was adopted as the criterion for statistical significance.

## Results

### Subjects

A total of 81 patients underwent the vestibular function test and the PSG test. Complete data were collected from 66 patients. Among them, 64 patients with AHI > 5/h were enrolled in the study group (128 ears; mean age 36.4 ± 7.1 years; range 20–50 years; female/male 7/57). The study group comprised 13 patients with mild OSA, 12 with moderate OSA, and 39 with severe OSA (Table 1).

**Table 1:** Demographic information and clinical characteristics of the OSA patients.

	***N***	**Mean ±SD**
Age	64	36.4 ± 7.1
**Gender**		
Female	7	–
Male	57	–
**BMI (kg/m** ^**2**^ **)**		
Overall	64	27.78 ± 4.24
Normal (18.5 ≤ BMI < 24)	13	22.28 ± 1.42
Early stage obesity (24 ≤ BMI < 28)	22	26.35 ± 1.05
Obesity (BMI ≥ 28)	29	31.32 ± 3.18
**AHI (/h)**		
Overall	64	45.25 ± 28.56
Mild (5 ≤ AHI < 15)	13	9.20 ± 3.12
Moderate (15 ≤ AHI < 30)	12	23.13 ± 5.04
Severe (AHI ≥ 30)	39	64.08 ± 19.46

In this study, 32 healthy volunteers (64 ears; mean age 36.2 ± 6.4 years; range 24–49 years; female/male 5/27) were recruited as a control group. There were no significant differences between the two groups in age, sex, or height.

### VEMPs Results

In the study group, subjects with bilateral oVEMP extraction were 38, unilateral oVEMP extraction were 15, and bilateral oVEMP disappearance were 11. And subjects with bilateral cVEMP extraction were 51, unilateral cVEMP extraction were 8, and bilateral cVEMP disappearance were 5, respectively. The response rates of oVEMP and cVEMP in the control group were 95.3 and 100.0%, respectively. In the study group, the response rate of oVEMP was 71.1%, and 85.9% of 128 ears had clear cVEMP, which were both significantly lower than that in the normal group. Comparing ARs of VEMPs in the study group with the controls, there was no statistical difference in oVEMP (26.03 ± 20.29% vs. 20.93 ± 13.75%, *t* = 1.132, *p* = 0.263) or cVEMP (26.84 ± 16.95% vs. 22.88 ± 17.32%, *t* = 0.903, *p* = 0.369).

Normal rates of oVEMP and cVEMP in these two groups were shown in Table 2. The results showed that the normal rates of VEMPs in the study group were significantly lower than those in the control group.

**Table 2:** Comparison of normal rates of vestibular function tests between the control group and the study group.

**Tests**	**Study group**		**Control group**		**χ^2^**	***p***
	***n***	**Normal rate**	***n***	**Normal rate**		
oVEMP	128	31.3%	62	95.2%	68.365	<0.001
cVEMP	128	26.6%	64	95.3%	80.675	<0.001
Caloric test	126	83.3%	64	93.8%	4.030	0.045
vHIT (Lateral)	128	100.0%	64	100.0%	N/A	N/A
vHIT (Anterior)	124	100.0%	64	100.0%	N/A	N/A
vHIT (Posteroir)	124	98.4%	64	100.0%	1.043	0.307

**Figure 1:**
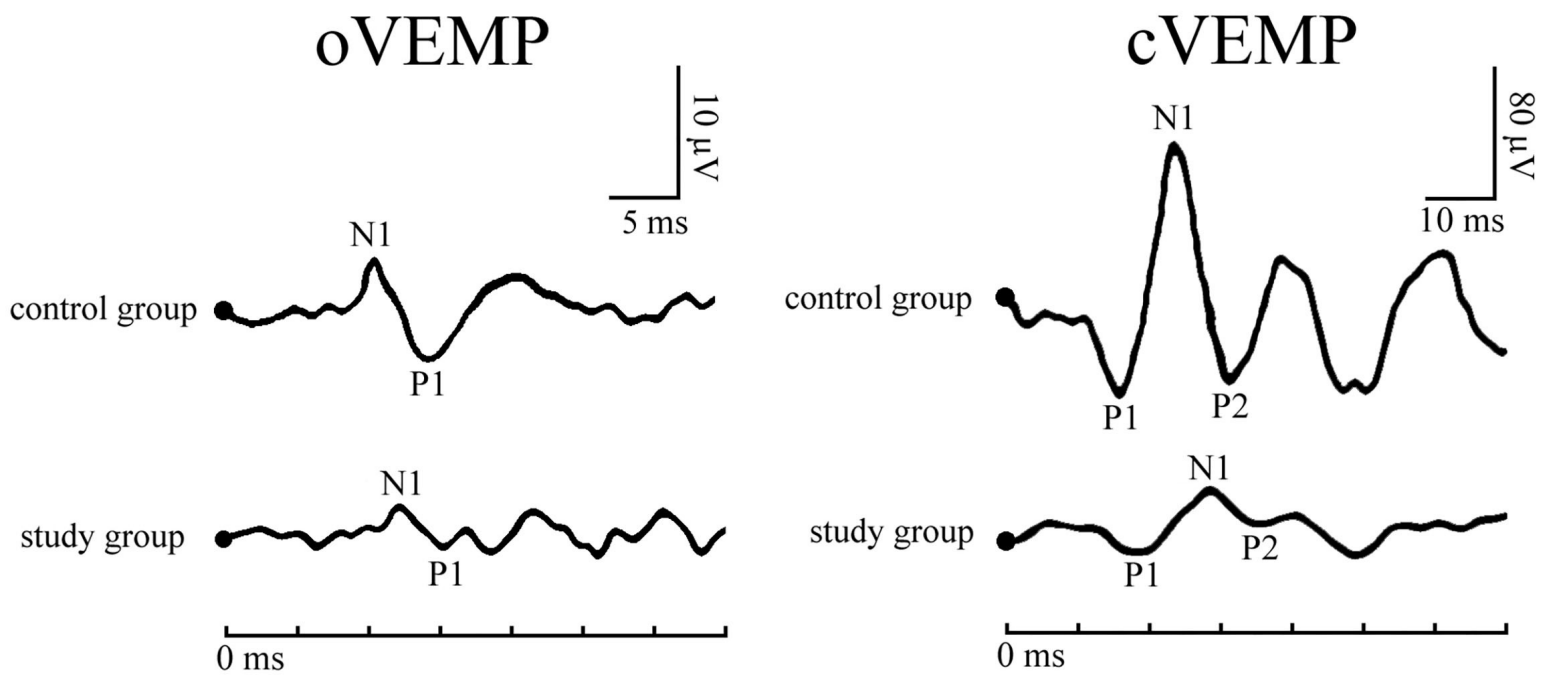
Typical vestibular-evoked myogenic potentials (VEMPs) waveforms of a patient with obstructive sleep apnea (OSA) and a healthy subject.

**Figure 2:**
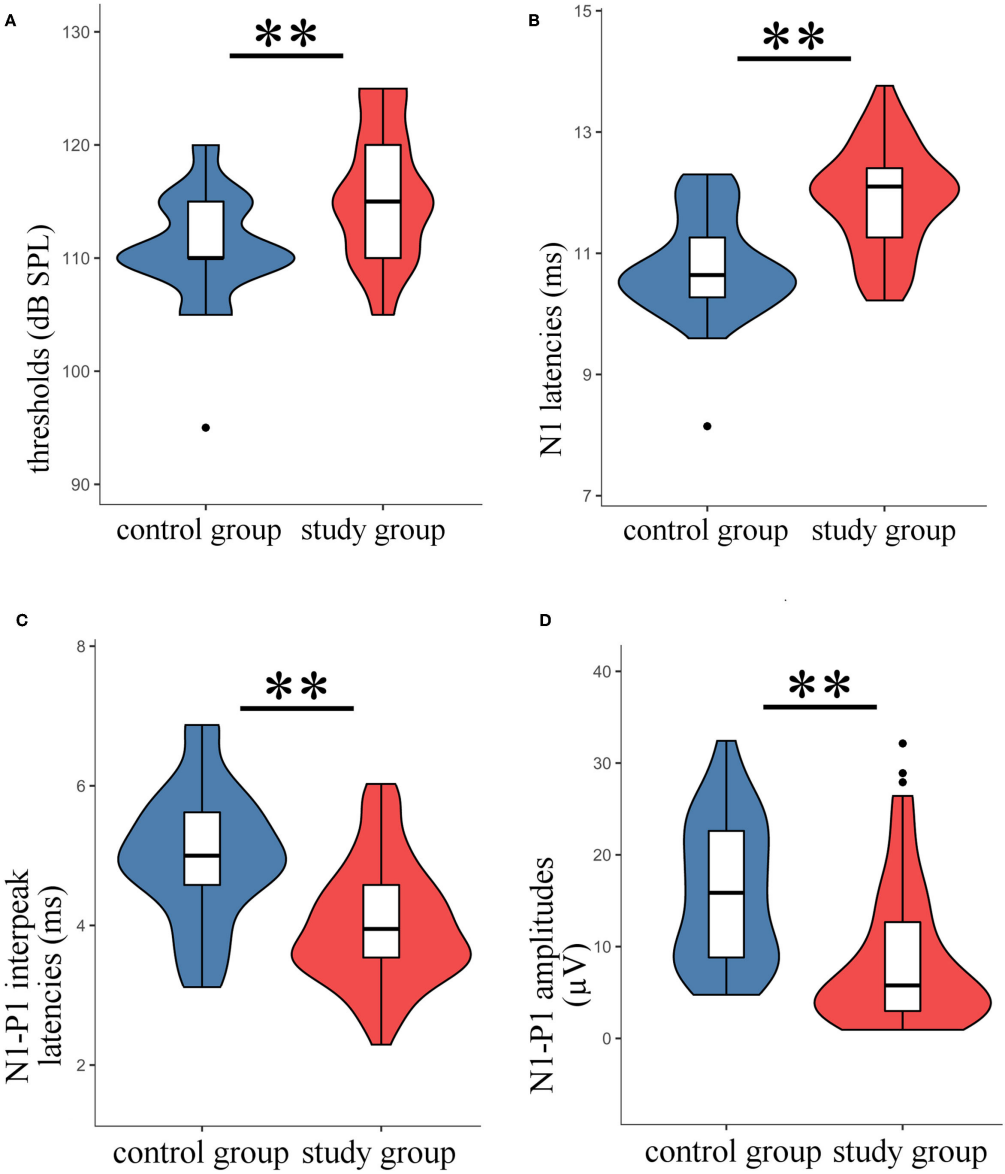
The oVEMP parameters in the study group were compared with those of the control group. The violin plot includes the box-whisker plot inside presenting the median, interquartile range (IQR), the upper limit (third quartile + 1.5 × IQR), and the lower limit (first quartile – 1.5 × IQR), and the violin outline presenting the density distribution (The mathematical predictions beyond the upper and lower limits were truncated because the boundary effect has no practical significance for this study.). **(A)** Thresholds; **(B)** n1 latencies; **(C)** n1-p1 interpeak latencies; **(D)** n1-p1 amplitudes; **t-test, *p* < 0.001.

**Figure 3:**
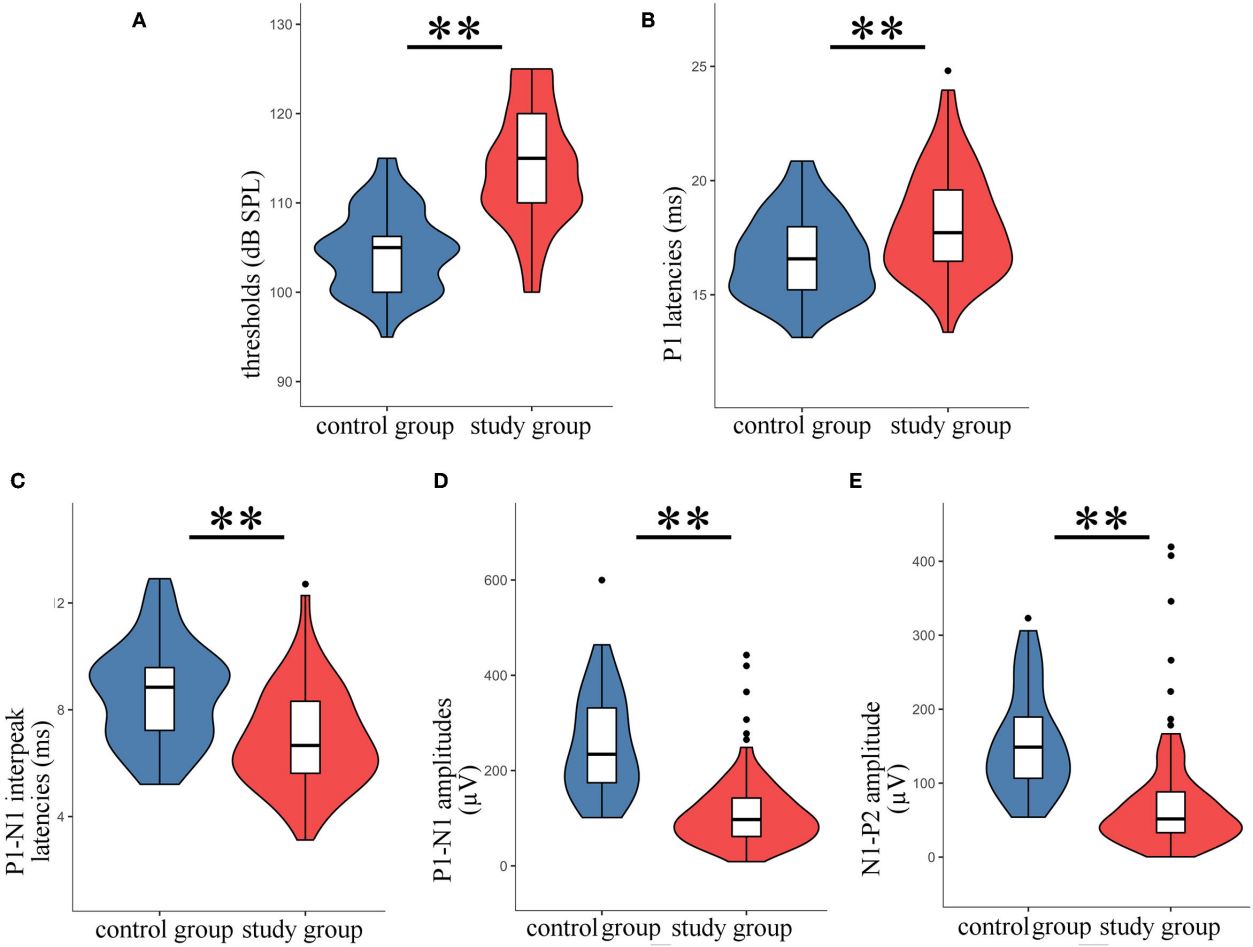
The parameters of cVEMP in the study group were compared with those of the control group. The violin plot includes the box-whisker plot inside presenting the median, IQR, the upper limit (third quartile + 1.5 × IQR), and the lower limit (first quartile – 1.5 × IQR), and the violin outline presenting the density distribution (The mathematical predictions beyond the upper and lower limits were truncated because the boundary effect has no practical significance for this study.). **(A)** Thresholds; **(B)** p1 latencies; **(C)** p1-n1 interpeak latencies; **(D)** p1-n1 amplitudes; **(E)** n1-p2 amplitudes; **t-test, *p* < 0.001.

Typical VEMPs waveforms of the control group and study group were shown in Figure 1. In the study group, elevated thresholds (*p* < 0.001), decreased n1-p1 amplitudes (*p* < 0.001), prolonged n1 latencies (*p* < 0.001), and shortened n1-p1 interpeak latencies (*p* < 0.001) in oVEMP were observed (Figure 2), while p1 latencies (*p* = 0.446) showed no significant change. With respect to cVEMP parameters, a series of deformations was discovered (Figure 3), namely, elevated thresholds (*p* < 0.001), decreased p1-n1 amplitudes (*p* < 0.001) and n1-p2 amplitudes (*p* < 0.001), prolonged p1 latencies (*p* < 0.001), and shortened p1-n1 interpeak latencies (*p* < 0.001). No significant changes in n1 latency (*p* = 0.488), p2 latency (*p* = 0.676), or n1-p2 interpeak latency (*p* = 0.179) were found.

### Caloric Test Results

A total of 126 ears in the study group (1 patient in the study group did not complete the caloric test for personal reasons) and 64 ears in the control group were examined. In the control group, 60 (93.8%) of 64 ears showed normal outcomes, and 4 ears showed canal paresis. In the study group, 105 (83.3%) of 126 ears showed normal outcomes, and 21 ears (16.7%) showed abnormalities. A significant difference in these parameters was found between the two groups (χ^2^ = 4.030, *p* = 0.045) (Table 2).

### vHIT Results

A total of 64 subjects in the study group underwent vHIT, 62 of whom completed all the examinations; only the test report of the LSC was obtained for the other 2 subjects due to personal reasons, while the reports of the ASC and PSC were not collected. All 32 subjects (64 ears) in the control group completed vHIT, and the results showed that the normal rate of each semicircular canal in the control group was 100.0%. Abnormalities were observed in 2 cases in the study group: 1 case had an abnormal gain value of the left PSC, and the other case had an abnormal gain value of the right PSC. In the vHIT results of the study group, the normal rate of the LSC was 100.0%, the normal rate of the ASC was 100.0%, and the normal rate of the PSC was 98.4%, all of which showed no significant difference compared with the control group (Table 2). The vHIT gain values of the two groups were also compared. There was no significant difference between the mean LSC gain value, the mean LSC 60-ms gain value, the mean ASC gain value, and the mean PSC gain value between the two groups.

### Results of Different Degrees of OSA

According to the AHI data, the study group was divided into three groups: mild, moderate, and severe. The normal rates and parameters of VEMPs, caloric test, and vHIT in multiple groups were compared (Table 3). In the cVEMP comparison results, n1-p2 amplitudes were found to be significantly different among the three groups. However, no significant difference was found in the oVEMP, caloric test, or vHIT.

**Table 3:** The normal rates and parameters of VEMPs, Caloric test, and vHIT in OSA of different severity.

**Items**	**Control group**		**Study group**						
			**Mild**		**Moderate**		**Severe**		***p***
	***n***	**Data**	***n***	**Data**	***n***	**Data**	***n***	**Data**	
AHI	32	2.49 ± 1.04	13	9.20 ± 3.12	12	23.13 ± 5.04	39	64.08 ± 19.46	<0.001
**oVEMP**									
Normal rate (%)	62	95.2	26	26.9	24	29.2	78	33.3	0.81
Threshold (dB SPL)	62	110.95 ± 4.72	13	114.62 ± 4.77	18	116.94 ± 5.18	60	115.00 ± 6.95	0.48
n1 latency (ms)	62	10.77 ± 0.83	13	11.66 ± 0.77	18	11.95 ± 0.99	60	11.94 ± 0.83	0.55
p1 latency (ms)	62	15.77 ± 1.04	13	16.08 ± 1.00	18	16.19 ± 1.54	60	15.81 ± 1.07	0.42
n1-p1 amplitude (μV)	62	16.00 ± 7.68	13	7.93 ± 6.66	18	8.17 ± 8.21	60	9.84 ± 9.57	0.67
**cVEMP**									
Normal rate (%)	64	95.3	26	38.5	24	12.5	78	26.9	0.12
Threshold (dB SPL)	64	104.67 ± 4.50	22	112.73 ± 7.83	20	113.25 ± 6.54	68	114.12 ± 6.69	0.68
p1 latency (ms)	64	16.70 ± 1.80	22	17.42 ± 2.05	20	17.99 ± 2.64	68	18.40 ± 2.28	0.22
n1 latency (ms)	64	25.33 ± 1.81	22	24.32 ± 2.49	20	25.10 ± 2.62	68	25.27 ± 2.28	0.27
p2 latency (ms)	64	30.32 ± 1.69	22	30.41 ± 2.49	20	30.59 ± 4.03	68	30.53 ± 2.66	0.98
p1-n1 amplitude (μV)	64	257.68 ± 112.06	22	151.46 ± 105.15	20	90.78 ± 54.01	68	109.12 ± 68.43	0.09
n1-p2 amplitude (μV)	64	155.27 ± 69.48	22	106.72 ± 98.49	20	54.72 ± 41.86	68	69.54 ± 67.42	0.036
**Caloric test**									
Normal rate (%)	64	93.8	26	76.9	22	81.8	78	85.9	0.49
SPV (50°C) (°/s)	64	20.81 ± 8.07	26	21.09 ± 13.76	22	19.75 ± 8.91	76	17.66 ± 11.94	0.41
SPV (24°C) (°/s)	64	15.12 ± 7.24	26	14.05 ± 7.37	22	14.87 ± 8.30	78	14.93 ± 9.43	0.90
**vHIT**									
Normal rate (L) (%)	64	100.0	26	100.0	24	100.0	78	100.0	–
Gain value (L)	64	1.05 ± 0.04	26	1.04 ± 0.09	24	1.04 ± 0.09	78	1.06 ± 0.13	0.68
60-ms gain value (L)	64	1.04 ± 0.05	26	1.00 ± 0.09	24	1.01 ± 0.14	78	1.02 ± 0.15	0.95
Normal rate (A) (%)	64	100.0	24	100.0	24	100.0	76	100.0	–
Gain value(A)	64	1.19 ± 0.12	24	1.12 ± 0.19	24	1.17 ± 0.25	76	1.21 ± 0.22	0.37
Normal rate (P) (%)	64	100.0	24	100.0	24	91.7%	76	100.0	–
Gain value (P)	64	1.18 ± 0.13	24	1.13 ± 0.21	24	1.16 ± 0.27	76	1.21 ± 0.26	0.50

*Mild OSA: 5/h ≤ AHI < 15/h; Moderate OSA: 15/h ≤ AHI < 30/h; Severe OSA: AHI ≥ 30/h. The normal rates were compared in study group by multi-group chi-square test. In the comparison of parameters, if the variance is homogeneous, then ANOVA is adopted; if the variance is not homogeneous, then H test is adopted. SPV, slow phase velocity. L, lateral semicircular canal; A, anterior semicircular canal; P, posterior semicircular canal*.

### Results of Different Degrees of BMI

No correlation between VEMPs parameters and BMI was found in the control group. To verify the consistency and further explore the impact of BMI on VEMPs in patients with OSA, the study group was divided into three groups normal BMI (18.5 ≤ BMI < 24 kg/m^2^), early-stage obesity (24 ≤ BMI < 28 kg/m^2^), and obesity (BMI ≥ 28 kg/m^2^) (Table 1), according to the WTO criteria of BMI classification for the Chinese population. The comparison of VEMPs, the caloric test, and vHIT of three groups is given in Table 4. Statistical differences were shown in the normal rate, n1 latency and p1 latency of oVEMP, and n1 latency and n1-p2 amplitude of cVEMP. No statistical difference was seen in the comparison of the caloric test or vHIT among the three groups.

**Table 4:** The normal rates and parameters of VEMPs, Caloric test, and vHIT in OSA patients with different degrees of BMI.

**Items**	**Normal BMI**		**Early-stage obesity**		**Obesity**		***p***
	***n***	**Data**	***n***	**Data**	***n***	**Data**	
BMI (kg/m^2^)	13	22.28 ± 1.42	22	26.35 ± 1.05	29	31.32 ± 3.18	<0.001
**oVEMP**							
Normal rate	26	50.0%	44	34.1%	58	20.7%	0.024
Threshold (dB SPL)	20	113.75 ± 5.59	29	115.86 ± 5.52	42	115.71 ± 7.21	0.290
n1 latency (ms)	20	11.21 ± 0.95	29	12.18 ± 0.75	42	12.04 ± 0.69	<0.001
p1 latency (ms)	20	15.19 ± 0.88	29	16.24 ± 1.20	42	16.06 ± 1.13	0.004
n1-p1 amplitude (μV)	20	11.58 ± 8.21	29	8.49 ± 6.92	42	8.63 ± 10.35	0.113
**cVEMP**							
Normal rate	26	42.3%	44	25.0%	58	20.7%	0.110
Threshold (dB SPL)	25	113.60 ± 6.85	34	114.71 ± 6.62	51	113.04 ± 7.08	0.533
p1 latency (ms)	25	17.502 ± 2.19	34	17.87 ± 2.00	51	18.61 ± 2.50	0.158
n1 latency (ms)	25	24.62 ± 2.55	34	24.24 ± 2.02	51	25.80 ± 2.36	0.012
p2 latency (ms)	25	29.77 ± 3.16	34	30.43 ± 2.81	51	30.93 ± 2.79	0.256
p1-n1 amplitude (μV)	25	135.42 ± 89.56	34	115.74 ± 75.36	51	102.88 ± 70.26	0.191
n1-p2 amplitude (μV)	25	82.87 ± 82.38	34	87.61 ± 69.84	51	61.19 ± 68.33	0.015
**Caloric test**							
Normal rate	26	76.9%	44	84.1%	56	85.7%	0.607
SPV (50°C) (°/s)	26	21.50 ± 12.67	44	18.77 ± 11.69	54	17.41 ± 11.62	0.170
SPV (24°C) (°/s)	26	16.72 ± 9.02	44	13.82 ± 7.46	56	14.54 ± 9.63	0.325
**vHIT**							
Normal rate (L)	26	100.0%	44	100.0%	58	100.0%	–
gain value (L)	26	1.08 ± 0.11	44	1.03 ± 0.09	58	1.05 ± 0.14	0.376
60-ms gain value (L)	26	1.03 ± 0.10	44	1.02 ± 0.13	58	1.01 ± 0.15	0.857
Normal rate (A)	26	100.0%	44	100.0%	54	100.0%	–
gain value (A)	26	1.21 ± 0.18	44	1.18 ± 0.23	54	1.16 ± 0.23	0.775
Normal rate (P)	26	100.0%	44	95.5%	54	100.0%	–
gain value (P)	26	1.19 ± 0.22	44	1.15 ± 0.29	45	1.07 ± 0.44	0.882

*Normal BMI: 18.5 ≤ BMI <24; Early-stage obesity: 24 ≤ BMI < 28; Obesity: BMI ≥ 28. The normal rates were compared by multi-group chi-square test. In the comparison of parameters, if the variance is homogeneous, then ANOVA is adopted; if the variance is not homogeneous, then H test is adopted. SPV, slow phase velocity. L, lateral semicircular canal; A, anterior semicircular canal; P, posterior semicircular canal*.

### VEMPs vs. Caloric Test vs. vHIT

The normal rates of oVEMP, cVEMP, caloric test, vHIT (lateral canal), vHIT (anterior canal), and vHIT (posterior canal) were calculated separately (Table 1). From the 6 test components above, 2 or 3 were randomly selected for intergroup comparison to explore whether the effects of OSA on various vestibular organs were synchronous or biased. Significant differences were found in the intergroup comparison of normal rates among the VEMPs, caloric test, and vHIT groups (*p* < 0.001), and no significant difference was found in other comparisons.

## Discussion

Obstructive sleep apnea, a common disorder associated with serious adverse health consequences, is attracting increasing attention from multidisciplinary studies seeking to improve the understanding of OSA complications and associated treatment. Researchers have sought to investigate the association of OSA with peripheral nerve injury and vestibular dysfunction (16, 17). It is speculated that the vascular endothelial damage and inflammation caused by hypoxemia due to sleep disruption and apnea may also lead to damage to vestibular end organs and their associated neural pathways. Mutlu et al. (17) reported a statistically significant deterioration of cVEMP: the response rate of the p1-n1 wave was 62.5% for the severe OSA group (35 ears) and 82.7% for the control group (43 ears). Ulusoy et al. (9) reported that the rate of obtaining waves of VEMPs in patients with moderate and severe OSA (85.0%) was significantly lower than that of the control group. However, Birk et al. (10) speculated that the VEMPs of patients with OSA were not significantly different from those of healthy people. In this study, the response rates of oVEMP and cVEMP in the study group were 71.1 and 85.9%, respectively. This demonstrated that patients with OSA would experience the VEMPs disappearance (2), reflecting that the utricle and saccule could be damaged.

Vestibular-evoked myogenic potentials may disappear in some patients with OSA, while there were still other patients whose VEMPs can be elicited. In the study by Mutlu et al. (17), there was a significant reduction in cVEMP amplitudes. Ulusoy et al. (9) discovered that in the moderate and severe OSAS groups, the p1-n1 amplitude and n1-p2 amplitude were lower than that in the mild OSAS groups. There are few observations of thresholds in the existing literature. In this study, significantly decreased amplitudes of oVEMP and cVEMP were also observed. Furthermore, it was found that the VEMPs threshold of the study group was significantly increased. In the authors' previous research, this phenomenon was collectively referred to as VEMPs impairment, which includes abnormalities in the threshold and amplitude, suggesting that the waveform is impaired even though VEMPs can still be elicited (5).

Even though reduced response rate, elevated threshold, and decreased amplitude were seen in VEMPs results of patients with OSA, the ARs of amplitude had no statistical difference compared with the control group. It showed that the unhealthy state of the vestibule in patients with OSA results in otolithic dysfunction that might be bilateral. Since the pathophysiological changes caused by OSA are systemic, it was reasonable that bilateral vestibular impairments resulting from OSA were synchronous and no difference. In previously published research, hearing loss (18), inner ear function (19), and brainstem auditory evoked potential (20) had been found bilateral impairments in patients with OSA, which was consistent with our findings of bilateral dysfunction of otolithic organs.

Body mass index is an important index in the diagnosis and treatment of OSA. If VEMPs results were correlated directly with BMI, the changes should be seen in the OSA population and normal controls. The patients with OSA in the study group were divided into three groups normal BMI, early-stage obesity, and obesity. Compared with the patients with OSA of normal BMI, the trends of change of VEMPs parameters in patients with early-stage obesity and obesity were different, or even opposite, which suggested that there may not be a linear correlation between VEMPs and BMI. And in the control group, no correlation between VEMPs parameters and BMI was found. Therefore, it was speculated that VEMPs tests were less disturbed by superficial soft tissue, such as fat, and were stable and reliable in testing the otolithic functions. It was the most likely reason that the changes of VEMPs could reflect impairments of vestibular organs and their conduction pathways resulting from OSA.

In the comparative analysis grouped by AHI, no significant relationship was found between severity of OSA and otolithic dysfunction except n1-p2 amplitude of cVEMP. Birk et al. (10) also found no clear correlation between VEMPs and AHI, which was consistent with this study. Ulusoy et al. (9) found an inverse relationship between the AHI value and the n1-p2 interval and p1-n1 amplitude values of cVEMP; however, no correlation with the AHI was found for other cVEMP parameters and all oVEMP parameters.

In this study, it was shown that the n1-p2 amplitudes of cVEMP in the moderate and severe patients with OSA were lower than the mild ones, but it cannot be explained with a clear meaning temporarily, because the sample size may be a limitation in this study. A significant decrease in otolithic function in patients with OSA has been reported by researchers, but no clear association between BMI/AHI and vestibular function was found in the existing studies. A larger number of investigations may provide further valuable information in future research.

In the existing literature, latency changes in oVEMP and cVEMP have rarely been reported. In this study, it was observed that the latency of the first wave (the first wave of the oVEMP waveform is n1, and the first wave of the cVEMP waveform is p1) showed a significant delay phenomenon, which was defined as the “delayed first wave (21)” of VEMPs. In the authors' previous studies of patients with severe deafness, typical changes such as threshold elevation and amplitude reduction were also found, but the delay in the “first-wave” latencies was not observed. OSA might cause damages not only to vestibular end organs but also affect conduction pathways such as the brain stem (21–23). Hypoxemia resulting from OSA has a negative impact on the neural structures such as the brainstem (23), which is an important neural pathway for the vestibular reflex system. In addition to the threshold and amplitude, the first wave latency of the VEMPs waveform may be another important parameter in defining peripheral nervous system lesions caused by systemic diseases.

In a review of the literature, few studies have examined changes in semicircular canal function in patients with OSA, and researchers have taken a different view. In the study by Gallina et al. (24), 27 out of 41 subjects (65.9%) showed unilateral or bilateral abnormalities in the caloric test, while only 1 out of 30 subjects (3.3%) showed abnormalities in the control group. Han et al. (25) found that the SPV of the caloric test was significantly different between the patients whose lowest oxygen saturation (LSaO_2_) was <50% and the patients whose LSaO_2_ was ≥ 80%. Birk et al. (10) found that among 42 patients with OSA having vHIT results, 7 had unilateral abnormalities and 3 had bilateral abnormalities. The study was not compared with the control group, but the authors found no correlation between PSG results and vHIT results. In this study, the normal rate of patients with OSA was indeed lower than that of the control group in the caloric test. In the vHIT, there was little difference in the results between the two groups. The results of VEMPs, caloric test, and vHIT showed that the vestibular organs and their nerve pathways were indeed impaired in patients with OSA, while the decrease in vestibular function was not synchronous but rather biased: the otolithic function was significantly changed, while the semicircular canal function was not. This phenomenon was considered to be related to multiple factors: (1) Nocturnal cerebral ischemia. Hypoxemia and reduced cerebral perfusion caused by repetitive apnea may lead to nocturnal cerebral ischemia in patients with OSA (26). The blood supply of otolithic organs is based on the vestibular branch of the labyrinth artery, which is a terminal artery (27). Due to the lack of collateral circulation, otolithic organs are sensitive to ischemia and hypoxia. Therefore, nocturnal cerebral ischemia in patients with OSA and scarce blood supply lead to ischemic injury of otolithic organs. (2) Ischemia-reperfusion. Circulation disorders may also cause repeated changes in endolymph pressure, resulting in changes in capillary pressure in the membranous labyrinth, which may lead to ischemia-reperfusion injury of otolithic organs (28). Ischemia-reperfusion leads to dysregulation of related oxidase, which damages the homeostasis of the otolith, further may cause otolith debris detaching from the macula of the otolith membrane (29, 30). (3) The blood supply of the semicircular canals is richer than that of the utricle and saccule. The labyrinthine artery bifurcates into the vestibular artery, which supplies the utricle, saccule, superior semicircular canal, and lateral semicircular canal. The stylomastoid artery, a branch of the posterior auricular artery, supplies the semicircular canals. Thus, otolithic organs may be more easily influenced than semicircular canals when hypoxemia and nightly oxygen desaturation caused by OSA occur. Therefore, this might be one of the main reasons that the oVEMP and cVEMP results of patients with OSA were worse than the caloric test and vHIT results. (4) Characteristics of diagnostic methods. In vHIT tests, the frequency of the head thrust stimulus was within physiological range to SCC, while sound/thermal stimulus to otolith was not within physiological range in VEMPs/Caloric test. Thus, the sensitivity of detecting potential abnormality might be higher in VEMPs/caloric test than in vHIT. The potential abnormality in VEMPs and caloric tests might be with less specificity of reflecting practical balance disorder at least in daily life, but they may still lead to practical health problems in the future. More diagnostic methods of vestibular function could be applied to further clarify the vestibular dysfunction of patients with OSA.

There were limitations in this study. The air caloric test was used to evaluate semicircular canal function, while it is less sensitive than the water caloric test and maybe underestimated for semicircular canal dysfunction. Moreover, corrected VEMP using an average of rectified background muscle activity could be applied to minimize the effects of muscle contraction fluctuation. We believe that the EMG rectification and upgrade of the equipment are important and more accurate trends may be observed after data correction. In terms of vestibular function diagnostic methods, more valuable results may be obtained by combining more diagnostic methods (the high-frequency acceleration head heave test, for instance). Besides, the sample size of the study group could be further increased to eliminate interference in the statistical results. Further work and data collection are still ongoing to verify the results or discover more scientific phenomena to explore.

## Conclusion

Obstructive sleep apnea-related specific changes in vestibular functions were found to be biased: utricular function and saccular function were affected more than semicircular canal function. These manifestations deserve much more attention from clinical health workers. More in-depth research on the pathogenesis of OSA-related vestibular dysfunction needs to be done to help physicians prevent vestibular complications and evaluate prognosis.

## Data Availability Statement

The raw data supporting the conclusions of this article will be made available by the authors, without undue reservation.

## Ethics Statement

The studies involving human participants were reviewed and approved by the Institutional Review Board of the Eye, Ear, Nose, and Throat Hospital of Fudan University. The patients/participants provided their written informed consent to participate in this study.

## Author Contributions

X-DX, B-JC, and A-RS designed the study, collected the data, wrote the manuscript, and did statistical analysis. H-PL and JY designed the study, collected the data, did statistical analysis, and modified the manuscript. QZ, YC, and D-DR collected the data. All authors contributed to the article and approved the submitted version.

## Funding

This study was supported by the National Natural Science Foundation of China (Nos. 81700915, 81771017, 81970880, 81970889, and 81970891); the Youth Foundation of Shanghai Municipal Health Commission (No. 20194Y0190); the Shanghai Rising Stars of Medical Talent Youth Development Program [Youth Medical Talents-Specialist Program, (2020) No. 87].

## Conflict of Interest

The authors declare that the research was conducted in the absence of any commercial or financial relationships that could be construed as a potential conflict of interest.

## Publisher's Note

All claims expressed in this article are solely those of the authors and do not necessarily represent those of their affiliated organizations, or those of the publisher, the editors and the reviewers. Any product that may be evaluated in this article, or claim that may be made by its manufacturer, is not guaranteed or endorsed by the publisher.
